# A Comparison of the Health Benefits of Customized Multivitamins and Standard Supplementation Post-bariatric Surgery: A Systematic Review

**DOI:** 10.7759/cureus.63253

**Published:** 2024-06-26

**Authors:** Mohamed F Zayed, Rana R Awis

**Affiliations:** 1 Pharmaceutical Sciences, Fakeeh College for Medical Sciences, Jeddah, SAU; 2 Clinical Nutrition, University of Nottingham, Nottingham, GBR

**Keywords:** customized multivitamins, sleeve gastrectomy, gastric bypass surgery, vitamin deficiencies, multivitamins, gastric weight loss surgery, bariatric surgeries

## Abstract

Rates of obesity increase worldwide year after year. This review explored if customized multivitamins (CMV) resulted in less micronutrient deficiency and higher serum levels of vitamins and minerals when compared to standard multivitamins (SMV) post-bariatric surgery in adults. Vitamins investigated were vitamins B1, B6, B_12_, D, parathyroid hormone (PTH), calcium, iron, hemoglobin, ferritin, folic acid, zinc, and magnesium. In Roux-en-Y gastric bypass (RYGB) patients weight loss surgeries (WLS) Forte or chewable CMV were studied, while in sleeve gastrectomy (SG) patients, WLS Optimum 1.0 (Opt. 1.0) or WLS Optimum 2.0 (Opt. 2.0) multivitamins were studied. An electronic search was performed on three databases (PubMed (n=28), Embase (n=120), and Cochrane (n=106)) to identify clinical trials and cohort studies. The inclusion criteria focused on studies since 2011 for adults ≥18 years old post-GB and SG. The keywords included bypass, sleeve, WLS, and multivitamins. Four clinical trials and three cohort studies were included. Jadad Scale was used to assess the quality and the bias risk in the clinical trials and the Newcastle-Ottawa scale (NOS) was used for the cohort studies. The PICO model and PRISMA rules were followed, where the outcomes targeted certain vitamin serum levels and the levels of deficiencies. The results of WLS Forte were better than SMV. The chewable CMV and Opt. 1.0 results were comparable to SMV. Opt. 2.0 was slightly better than Opt. 1.0. Further modifications would enhance the CMV presented in this systemic review. SMV would still be recommended until CMV are modified and tested. Multi-center trials that monitor the effect of the modified CMV on the serum levels of vitamins and minerals in the longer term in different wider populations are needed.

## Introduction and background

The prevalence of morbid obesity worldwide and related chronic diseases has increased and is predicted to increase [1,2]. Based on the World Health Organisation (WHO) in 2016, approximately 13% of the adult population worldwide were obese with body mass index (BMI) ≥30 kg/m^2^ [3]. In the morbidly obese population (BMI >40 kg/m^2^), lifestyle modifications and medications have all failed to achieve long-term weight loss [4,5]. The failed attempts to lose weight led to weight loss surgeries (WLS) or bariatric surgeries to become a more convenient solution [6,7]. The National Institute of Health and Care System (NICE) recommends bariatric surgery for patients with a BMI ≥40 kg/m^2^, or a BMI ≥35 kg/m^2^ to 40 kg/m^2^ with a chronic illness such as type 2 diabetes or high blood pressure, that could be improved if the weight is reduced [8]. Roux-en-Y gastric bypass (RYGB) and sleeve gastrectomy (SG) were the most performed bariatric surgeries [9,10]. In the SG, 80% of the stomach is removed, while in the RYGB a gastric pouch of 10 to 30 mL is attached directly to the middle part of the intestine, the proximal jejunum [11]. Both surgeries result in modifications in the capacity of absorption, in the amount of acid present in the gastrointestinal tract (GIT), in the motility, in the hormones produced, and in the microflora present in the gut [12]. The surgeries have many beneficial effects including mainly weight loss and remission of some chronic diseases [13,14]. Yet with a caloric-deficient diet and malabsorption of many nutrients, both surgeries have a dangerous and common side effect, which is vitamin and mineral deficiency [13-16]. Some of the frequently experienced deficiencies post-bariatric surgeries and their impact on the long-term are mentioned in Table 1 [17-23]. The normal serum levels for the vitamins and minerals of concern are mentioned in Table 2 [20-23]. The focus of this systemic review will be vitamins and minerals that are known to cause deficiencies after both RYGB and SG [24,25].

**Table 1:** The most frequently noted vitamin and mineral deficiencies, their prevalence post-bariatric surgeries, and the signs of deficiency. PTH, parathyroid hormone; RYGB, Roux-en-Y gastric bypass; SG, sleeve gastrectomy; TIBC, total iron binding capacity

Vitamin/mineral	Prevalence	Signs of deficiency
Vitamin B1	From 1% up to 49% [17]	Early deficiency signs and symptoms: dry or wet beriberi, gastroenterological symptoms: nausea, vomiting, slow gastric emptying, constipation, and jejunal dilation. Advanced deficiency signs and symptoms: Wernicke’s encephalopathy
Vitamin B_12_	RYGB: <20%, SG: 4% to 20% [17]	Early deficiency signs and symptoms: pernicious anemia, megaloblastic anemia, numbness and tingling in extremities (due to nerve damage), tinnitus, palpitation, and shortness of breath. Advanced deficiency signs and symptoms: angina, distorted mental status
Folate and folic acid	Up to 65% [18]	Pigmentation changes; ulcers in the skin and oral mucosa
Iron	RYGB: 20% to 55%, SG: <18% associated with elevated TIBC [18]	Microcytic anemia, fatigue, palpitations, lower immunity function
Vitamin D and calcium	Up to 100% associated with elevated PTH levels and decreased calcium levels (hypocalcemia) [19]	Cramps in the legs, osteoporosis, osteomalacia, calcium deficiency, and weakness in muscles
Vitamins A	RYGB only: up to 70% [20]	Night blindness, loss of taste, and poor wound healing
Zinc	RYGB: up to 40%, SG: up to 19% [21,22]	Early deficiency signs and symptoms: infertility, decreased immunity, and increased rate of infection. Advanced deficiency signs and symptoms: hair loss, poor wound healing, and night blindness
Copper	RYGB: between 10% to 20%; SG: one case reported [23]	Early deficiency signs and symptoms: hypochromic anemia, pancytopenia, neutropenia, hair, nails, and skin hypopigmentation, high cholesterol levels, and bone metabolism biomarkers distorted. Advanced deficiency signs and symptoms: abnormal gait

**Table 2:** The normal serum levels of vitamins and minerals investigated in this systemic review.

Vitamin or mineral (unit)	Normal serum level
Calcium (mmol/L)	2.10-2.55
Ferritin (mcg/L)	20-200
Folic acid (nmol/L)	9.0-36.0
Hemoglobin (mmol/L)	Male: 8.4-10.8
Hemoglobin (mmol/L)	Female: 7.4-9.9
Iron (mmol/L)	9.0-31.0
Magnesium (mmol/L)	0.71-0.93
Parathyroid hormone (pmol/L)	1.3-6.8
Vitamin B1 (nmol/L)	95-175
Vitamin B6 (nmol/L)	25-100
Vitamin B_12_ (pmol/L)	150-640
Vitamin D (nmol/L)	>50
Zinc (mcmol/L)	9.2-18.4

The risk of iron, ferritin, and vitamin B_12_ deficiencies was increased after RYGB and SG surgeries, due to the reduced acid (hydrochloric acid) production, decreased intrinsic factor secretion in the GIT, the use of proton-pump inhibitors, and the inability to intake food rich in iron [16,17]. Vitamins B1 and B6 follow the example of vitamin B_12_ and iron as they all need gastric acids to cleave them from dietary proteins [5,14]. After RYGB, the incidence of deficiencies in vitamin B_12_, folic acid, and iron were 50%, 15%, and 66%, respectively [16]. Owing to the vitamin D production mechanism, when the skin gets exposed to sunlight, obesity was thought to be a risk factor for vitamin D deficiency along with the bypass of the proximal small intestine in the RYGB where it is usually absorbed [5,16]. PTH levels were important as they were linked to calcium and vitamin D; when PTH levels rise, calcium resorption occurs, and vitamin D formulation is stimulated [5]. Zinc undertakes a key role in wound healing and also impacts albumin levels [17,18]. The British Obesity and Metabolic Surgery Society (BOMSS) specified general guidelines to avoid deficiencies after bariatric surgeries including daily multivitamin supplementation, injection of vitamin B_12_ every three months, additional vitamin D and calcium supplementation, iron starting with 200 mg once a day and could increase the dose if a patient was a woman during menstruation age, and leaving two hours between iron and calcium supplements to avoid one impacting the other’s absorption [14]. The American Society for Metabolic and Bariatric Surgery (ASMBS) advises that post-RYGB and GS surgeries patients need to be supplemented with the following multivitamins shown in Table 3 to avoid deficiencies [13,19].

**Table 3:** The vitamins and minerals recommended by the ASMBS post-RYGB and GS surgeries. The ASMBS guidelines [13,19]. ASMBS, American Society for Metabolic and Bariatric Surgery; RYGB, Roux-en-Y gastric bypass; SG, sleeve gastrectomy

Vitamin B1	Daily oral 12 mg. A 50 mg in a B-complex or a multivitamin would be better, and no upper limit as it has low toxicity potential
Vitamin B_12_	Daily oral 350 mg to 500 mg; no upper limit as it has low toxicity potential
Folic Acid	Daily 400 mg to 800 mg in the oral multivitamin, female of childbearing age: daily oral 800 to 1000 mg
Iron	Males or females, who stopped menstruation or those without a history of anemia: At least daily 18 mg in the oral multivitamin. Women during menstruating age: at least 45 to 60 mg daily of elemental iron. Taken apart from acid-reducing medications, calcium supplements, and foods containing polyphenols or phytates
Vitamin D and calcium	Calcium: daily 1200 to 1500 mg, vitamin D3: daily oral 3000 IU
Vitamins A, E, and K	Vitamin A: daily 5000 to 10000 IU, vitamin K: daily 90 to 120 mcg, vitamin E: daily 15 mg
Zinc	RYGB: daily 8 mg to 22 mg in a multivitamin, SG/LAGB: daily 8 mg to 11 mg in a multivitamin

It has been noted that due to several issues, patients were not compliant with the recommended vitamin and mineral supplementation [20-22]. Adherence to several pills like multivitamins and additional elemental supplements, in a day with gaps in between proved to be hard [20-22]. Patients found it either hard to swallow, hard to remember, or expensive [21,22]. There was a significant decline in patients’ compliance with multivitamins (P<0.001), vitamin D (P<0.001), and calcium (P=0.002) between year one and year four post-surgery [21,22]. Physiological symptoms due to the surgery like bloating, gastroesophageal reflux, and nausea were recognized as one of the main reasons as well [22]. Additionally, some studies argue that SMV were insufficient to prevent deficiencies and some of the doses in the guidelines were insufficient too [8,15]. Gesquiere et al. noticed that with SMV supplementation, there was still a significant decrease in hemoglobin, ferritin, vitamin B_12_, and zinc levels [8]. Gasteyger et al. reported that 60% of 137 patients needed more than one supplement within two years of the surgery [15]. One CMV that provide optimized doses of all vitamins and minerals post-bariatric surgeries would result in lower deficiencies, better serum levels, and better adherence. There have not been any systematic reviews that investigated if the currently studied CMV would be of more benefit to adults’ post-bariatric surgeries compared to SMV. The aim of this systemic review would be to evaluate if CMV would result in fewer deficiencies and better serum levels of vitamins and minerals when compared to SMV in adults’ post-bariatric surgeries. 

## Review

Methodology (search strategy)

The PICO model has been used to assist in clearly defining the research question: "Will using customized multivitamin supplementation post-gastric bypass and SG be of more benefit when compared to nonusers or standard multivitamin users?" The PICO model that was utilized is described in Table 4.

**Table 4:** The PICO model that was utilized in this study. CMV, customized multivitamin; SMV, standard multivitamins

Population: adults >18 post-bariatric surgery
Intervention: CMV.
Comparison: CMV group versus SMV group, not taking any supplement or stopped taking CMV midway (nonuser group).
Outcome: the serum concentration of vitamins and minerals as well as deficiencies encountered, if mentioned.
Types of studies included - clinical trials and cohort studies.

**Figure 1:**
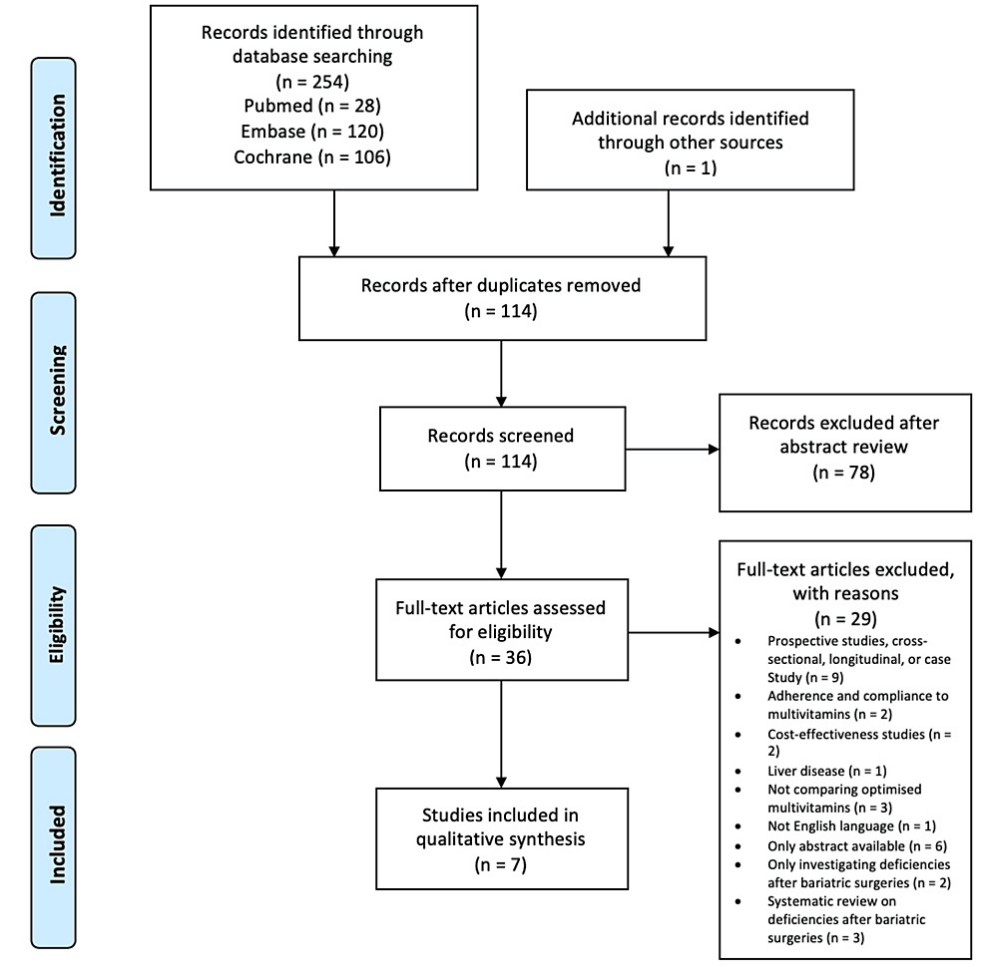
A PRISMA flow diagram that explains the databases used, the number of studies found in each database, the number of excluded studies, and the number of selected studies. PRISMA, Preferred Reporting Items for Systematic Reviews and Meta-Analyses

To conduct an electronic search, databases that are recognized for their comprehensive and versatile content in relation to medicine, nutrition, and surgery were selected: PubMed (n=28), Embase (n=120), and Cochrane (n=106). The total number of studies found was 254. The availability of studies related to bariatric surgeries was limited due to the surgeries being recent [23,24]. The first RYGB and SG performed were in 1977 and 2005, respectively [24,25]. Preferred Reporting Items for Systematic Reviews and Meta-Analyses (PRISMA) 2009 has been used in the search strategy to identify the studies that would be selected in the review [25]. PRISMA 2009 was chosen instead of the 2020 version as it was more relevant to the review due to the absence of registers and reports [26]. The studies generated were screened for duplicates and checked if they were relevant to the research question through the title and abstract screening. A full-text review of the remaining 36 studies was done to assess the eligibility by applying the inclusion and exclusion criteria. After the review had been completed, four trials and three cohort studies were included. The remaining 247 studies were excluded as they were not eligible for inclusion. The PRISMA flow diagram (Figure 1) demonstrates the results of the search and the reasons for the exclusion of other studies in more detail [26]. The terms mentioned in Table 5 have been combined with Boolean terms "OR" and "AND" to conduct the search on the three databases. The keywords used included medical subject heading (MESH) terms and free texts.

**Table 5:** The terms used during the search strategy on databases: Pubmed, Embase, and Cochrane. RYGB, Roux-en-Y gastric bypass; SG, sleeve gastrectomy; WLS, weight loss surgeries

Procedure	Intervention
Gastric bypass	Multivitamin supplementation
Bypass	Multivitamin
Sleeve	Multivitamins
SG	WLS
Bariatric surgery	Optimized multivitamins
RYGB	WLS Forte OR WLS Optimum

Inclusion and exclusion criteria

The inclusion criteria were mainly focused on studies with adult participants above the age of 18 years old, who have had bariatric surgeries, either gastric bypass or SG. All selected papers were in the English language and peer-reviewed. Clinical trials and cohort studies were chosen in the inclusion criteria as they compared the intervention CMV to the SMV or nonuser. In this way, a definite answer to the research question could be reached. Studies before 2011 were excluded to ensure a review of the contemporary evidence base. Any type of study other than a clinical trial or cohort study was excluded.

Quality assessment

With respect to the quality and risk assessment of the included studies in this systemic review, two different scales were used. The Jadad scale for assessing the quality of the four clinical trials and the Newcastle-Ottawa scale (NOS) for evaluating the quality of the three cohort studies [27-29].

Jadad scale

The Jadad scale takes into consideration essential aspects, which are tests of reliability, bias risk, and validity [28]. The scale total score ranges between 0 and 5, (very poor quality - excellent quality), assessed through a list of seven questions [28,30].

The Newcastle-Ottawa scale

The NOS star system assesses three different criteria, which are the study groups’ selection, the groups’ comparability, and the outcome of the study [29]. Each element in the selection and outcome sections could obtain a maximum of one star; however, the comparability element could obtain a maximum of two stars [29]. This would lead to a maximum score of nine stars for a study, where a score ≥7 stars reflects the high quality and low risk of bias [29,31].

Results of quality assessment

Based on the Jadad scale calculator, out of the four assessed trials, two were found to be of excellent quality and low risk of bias, one was found to be of good quality, and one was found to be of poor quality. Dogan et al. and Heusschen et al. description of the randomization and the double-blinding processes were appropriate and clear [16,17]. Perin et al. trial had a defined randomization process, but the trial was not blinded [32]. The author justified that blinding did not affect the outcome, as the primary outcome was an objective measure [32]. Heusschen et al. trial was a single-arm open-label study with no randomization or double-blinding [33]. The researchers used the results of their previous trial, included, and compared it to their later study instead of using a control [17,33]. The later trial was considered as a continuation of the earlier work [17,33]. The groups included in both trials were of similar characteristics and the researchers, surgeons, hospital, and technique were similar too, which was the main reason for the inclusion of the later trial even though it was found to be of poor quality [17,33]. The calculation of the scores for the Jadad scale for each study is detailed in Table 6.

**Table 6:** The Jadad scale scores of quality assessment for each study used.

Study	Item 1	Item 2	Item 3	Item 4	Item 5	Item 6	Item 7	Total score
Dogan et al. [16]	1	1	1	1	1	0	0	5
Perin et al. [32]	1	1	0	0	1	0	0	3
Heusschen et al. [17]	1	1	1	1	1	0	0	5
Heusschen et al. [33]	0	0	0	0	1	0	0	1

Using NOS, it was found that all three were of high quality and low risk of bias. Mainly all studies have shown good study group selection, where different groups were fair representative of the average community and were chosen from the same community. In the comparability section, the confounding factors were accounted for in the Homan et al. and Smelt et al. studies but not in the Schijns et al. study [5,6,34]. The percentage of participants lost to follow-up was not mentioned in the Schijns et al. study, and the justification for the mentioned percentage of loss was not explained in the Smelt et al. study [5,6]. A summary of the stars achieved in each section and overall is mentioned in Table 7.

**Table 7:** The results of the NOS star system for each section and the total score for the three included cohort studies. NOS, Newcastle-Ottawa scale

Study	Selection	Comparability	Outcome	Total stars achieved
Homan et al. [34]	4 Stars	2 Stars	3 Stars	9 Stars
Schijns et al. [5]	4 Stars	1 Star	2 Stars	7 Stars
Smelt et al. [6]	4 Stars	2 Stars	2 Stars	8 Stars

Studies’ results

Out of the seven included studies, four studies focused on patients post-RYGB, and three studies focused on patients’ post-SG [5,6,17,33-34]. One trial investigated the effectiveness and safety of CMV "WLS Forte" in relation to SMV in RYGB patients; the same trial was conducted for SG patients using Opt. 1.0 as the CMV [16,17]. Homan et al. cohort study was a continuation of the randomized controlled trial (RCT) conducted by Dogan et al. [16,34]. The cohort study looked into "WLS Forte" effectiveness and safety for a longer term of three years in the same patients’ groups [34]. Another larger-scale cohort study looked at the effectiveness of CMV "WLS Forte" in RYGB surgery patients during the initial three years [5]. A chewable CMV with better absorption was studied by Perin et al. in comparison with SMV after RYGB surgery [32]. Smelt et al. performed a large-scale cohort study to explore the effectiveness of CMV "Opt. 1.0" in SG patients over a longer term of five years [6]. Heusschen et al. compared the effectiveness of two versions of CMV, Opt. 2.0 versus the previously studied Opt. 1.0 in an open-labeled trial in patients who underwent SG [33].

Participants’ basal disposition

There were 2697 participants, respectively, all of which were adults ≥18 years old and less than 65 with BMI ≥35 kg/m^2^. The majority of participants were females [5,6,16,17,14-34]. The general characteristics of the studies and the participants are included in Table 8.

**Table 8:** Key elements of the studies and general characteristics of participants in each study. WLS, weight loss surgery; SMV, standard multivitamin; RCT, randomized controlled trial; SD, standard deviation; PTH, parathyroid hormone; SG, sleeve gastrectomy

Studies	Number of participants	Type of study/country/duration/time serum measurements taken	Mean age/BMI	Type of surgery/intervention	Vitamin/mineral deficiencies studied
Dogan et al. [16]	148 Participants	Type of study: RCT; country: Netherlands; duration: 1 year time; serum measurements taken: baseline, at 6th month and 1st year	Mean age, yr±SD: SMV: 43.4±10.0, WLS Forte: 45.3±10.2 (P=0.26); mean BMI, Kg/m^2^±SD: SMV: 44.8±4.8; WLS Forte: 44.8±6.4 (P=1.00)	RYGB/ WLS Forte vs. SMV	Vitamin B_12_, vitamin D, folic acid, PTH, calcium, zinc, magnesium, and iron
Homan et al. [34]	148 Participants	Type of study: cohort; country: Netherlands; duration: 3 years time; serum measurements taken: baseline, and at 36th month	Mean age, yr±S.D: SMV: 46±12.0; WLS Forte: 44±9; none: 43±10; mean BMI, Kg/m^2^±SD: SMV: 45±6; WLS Forte: 44±5; none: 46±5	RYGB/WLS Forte vs. SMV and nonuser (not taking any supplement)	Vitamin B_12_, vitamin D, PTH, calcium, zinc, magnesium, and iron
Perin et al. [32]	56 Participants	Type of study: RCT; country: United States; duration: 6 months time; serum measurements taken: baseline, at 3rd and 6th month	Mean total age, yr±SD: 43.1±10.2; mean total BMI, Kg/m^2^±SD: 46.2±7.2	RYGB/Chewable CMV vs. SMV	Vitamins A, vitamin B_12_, vitamin D, vitamin E, thiamine, iron, iron-binding capacity, iron saturation, calcium, and PTH
Schijns et al. [5]	1160 Participants	Type of study: Cohort Country: Netherlands Duration: 3 years Time Serum Measurements Taken: Baseline, at 1st, 2nd, and 3rd year	Mean age, yr: ≥18 to 60; mean BMI, Kg/m^2^±SD: user: 44.1±5.4; Nonuser: 44.4±5.3	RYGB/WLS Forte vs. nonuser (stopped taking CMV)	Vitamin D, vitamin B_12_, folic acid, folate, ferritin, and PTH
Heusschen et al. [17]	139 Participants	Type of study: RCT; country: Netherlands; duration: 1 year time; serum measurements taken: baseline, at 6th month and 1st year	Mean Age, yr±S.D: SMV: 39.7±10.8; WLS Forte: 38.2±12.4; mean BMI, Kg/m^2^±SD: SMV: 48.4±9.9; WLS Forte: 47.6±9.0	SG/WLS Optimum 1.0 vs. SMV	Vitamin B_12_, vitamin D, folic acid, PTH, calcium, zinc, magnesium, and iron
Smelt et al. [6]	970 Participants	Type of study: cohort; country: Netherlands; duration: 5 years time; serum measurements taken: baseline, at 6th, 12th, 24th, 36th, and 48th months	Mean age, yr±SD: SMV: 43±11; WLS Optimum: 46±10 (P=0.001); mean BMI, Kg/m^2^±SD: nonuser: 44±6; user: 43±5 (P=0.011)	SG/WLS Optimum 1.0 vs. nonusers (not taking CMV)	Vitamin B_12_, vitamin B1, vitamin B6, vitamin D, iron, ferritin, hemoglobin, albumin, calcium, and PTH
Heusschen et al. [33]	76 Participants	Type of study: open-label trial; country: Netherlands; duration: 1 year time; serum measurements taken: baseline, at 6th month and 1st year	Mean age, yr±SD: WLS Optimum 1.0: 38.2±12.4; WLS Optimum 2.0: 38.1±12.9; mean BMI, Kg/m^2^±S.D: WLS Optimum 1.0: 47.6±9.0; WLS Optimum 2.0: 47.1±7.9	SG/WLS Optimum 2.0 vs. Optimum 1.0	Vitamin B_12_, vitamin D, folic acid, PTH, hemoglobin, ferritin, calcium, zinc, and magnesium

Serum concentration of vitamins and deficiencies

All studies mainly focused on the serum concentration of vitamin B_12_, vitamin D, folic acid, and PTH [5,6,16,17,32-34]. Ferritin and vitamin B1 were measured in all the previous studies except one [33]. Five looked at vitamin B6 and calcium [6,16,17,33,34]. Zinc and magnesium were measured in four studies [16,17,33,34]. The times when measurements of serum levels were taken are mentioned in Table 9. The concentration of these vitamins and minerals in each CMV is mentioned in Table 9. The serum levels for each vitamin and mineral encountered by the consumption of each CMV, SMV, or non-consumption of either are mentioned in Tables 10, 11.

**Table 9:** The concentration and the recommended dietary allowance in each CMV included in the review. Conc., concentration; RDA, recommended dietary allowance; IU, international unit

Ingredients	WLS Forte (Conc., RDA%)	Investigational chewable (Conc.)	WLS Optimum 1.0 (Conc., RDA%)	WLS Optimum 2.0 (Conc., RDA%)
Calcium (mg)	-	1500	-	-
Folic acid (mcg)	600, 300%	800	300, 150%	500, 250%
Iron (mg)	70, 500%	36	21, 150%	28, 200%
Magnesium (mg)	-	50	30, 8%	-
Vitamin B1 (mg)	2.75, 250%	12.5	2, 182%	2.75, 250%
Vitamin B6 (mg)	2.8, 200%	4	2, 143%	2, 143%
Vitamin B_12_ (mcg)	350, 14000%	500	10, 400%	100, 4000%
Vitamin D (mcg)	12.5, 250%	1500 IU	7.5, 150%	7.5, 150%
Zinc (mg)	22.5, 225%	15	15, 150%	28, 280%

**Table 10:** Results' details presented in the study. Data presented as mean±SD. *P≤0.05 or P<0.01 or P<0.001 CMV, customized multivitamin; PTH, parathyroid hormone; SMV, standard multivitamin; SD, standard deviation; nonuser: not taking any supplement

Study	Calcium (mmol/L)	Ferritin (mcg/L)	Folic acid (nmol/L)	Hemoglobin (mmol/L)	Iron (mmol/L)	Magnesium (mmol/L)
Normal values	2.10-2.55	20-200	9.0-36.0	Female: 7.4-9.9; male: 8.4-10.8	9.0-31.0	0.71-0.93
Dogan et al. [16], Δ0-12 months	SMV: +0.009±0.21; CMV: +0.001±0.09, P=0.77	SMV: -18.4±61.8; CMV: +4.9±81.3, P=0.08	SMV: +5.7±7.3; CMV: +11.2±11.9, *P=0.002	SMV: +0.029±0.7; CMV: -0.063±0.5, P=0.64	SMV: +2.9±6.9; CMV: +4.5±5.2, P=0.14	SMV: +0.11±0.52; CMV: +0.03±0.05, P=0.25
Dogan et al. [16]. After 12 months	SMV: 2.32±0.21; CMV: 2.32±0.10, P=1.00	SMV: 80.8±71.0; CMV: 108.3±83.1, *P=0.05	SMV: 22.9±8.0; CMV: 29.3±11.5, *P<0.001	SMV: 8.5±0.8; CMV: 8.6±0.7, P=0.50	SMV: 16.0±5.3; CMV: 16.8±5.5, P=0.42	SMV: 0.89±0.52; CMV: 0.81±0.06, P=0.29
Homan et al. [34]. After 3 years	SMV: 2.34±0.02; CMV: 2.35 ±0.02; None: 2.43±0.04, P>0.05	SMV: 85±9; CMV: 117±9; none: 85±13; *P (CMV vs. SMV) <0.001; *P (CMV vs. nonuser)=0.047; P (SMV vs. nonuser)=1.000	SMV: 22.7±0.9; CMV: 32.7±0.9; none: 15.6±1.7, *P (CMV vs. SMV)<0.001, *P (CMV vs. nonuser)<0.001, *P (SMV vs. nonuser)<0.001	SMV: 8.4±0.1; CMV: 8.6±0.1; none: 8.1±0.1, *P (CMV vs. SMV)=0.050, *P (CMV vs. nonuser)=0.003, P (SMV vs. nonuser)=0.202	Not mentioned	SMV: 0.86±0.03; CMV: 0.83±0.03; none: 0.82±0.06, P>0.05
Perin et al. [32]. After 6 months	Not mentioned	Not mentioned	Not mentioned instead Folate (ng/mL), Normal range: 5.3-24; SMV: 18.8±7.2; CMV: 20.0±5.7, P=0.526	Not mentioned	Iron (mcg/dL), normal range: 67-185; SMV: 71.5±32.7; CMV: 59.5±50.2, P=0.607; Iron-binding capacity (mcg/dL), normal range:250-450; SMV: 325.5±64.9; CMV: 236.0 (−), P=0.286; Iron saturation (%), normal range: 26-39; SMV: 22.0±6.2; CMV: 21.0±7.1, P=0.845	Not mentioned
Schijns et al. [5]. Δ0-3 years	Not mentioned	CMV: -24±71; nonuser: -42±71, P>0.05	CMV: +18±11; nonuser: +7±11, *P≤0.05	Not mentioned	Not mentioned	Not mentioned
Schijns et al. [5]. After 3 years	Not mentioned	CMV: 77±80; nonuser: 74±53, P>0.05	CMV: 34±11; nonuser: 23±11, *P≤0.05	Not mentioned	Not mentioned	Not mentioned
Heusschen et al. [17]. Δ0 – 12 months	SMV: +0.03±0.10; CMV: +0.05±0.11, P>0.05	SMV: -3.61±80.05 CMV: +2.65±60.33, P>0.05	SMV: +2.42±7.05; CMV: +6.84±9.73, *P≤0.05	SMV: -0.20±0.54; CMV: -0.34±0.66, P>0.05	SMV: +4.90±6.20; CMV: +4.56±5.64, P>0.05	SMV: +0.03±0.06; CMV: +0.03±0.07, P>0.05
Heusschen et al. [17]. After 12 months	SMV: 2.38±0.07; CMV: 2.40±0.08, P>0.05	SMV: 130.5±75.9; CMV: 150.0±116.5, P>0.05	SMV: 19.6±6.6; CMV: 24.4±10.3, *P≤0.05	SMV: 8.5±0.7; CMV: 8.5±0.6, P>0.05	SMV: 16.1±7.0; CMV: 15.1±5.3, P>0.05	SMV: 0.83±0.04; CMV: 0.83±0.06, P>0.05
Smelt et al. [6]. Δ0-48 months	CMV: -0.05±0.12; nonuser: -0.02±0.12, P=0.15	CMV: -11.5±148.6; nonuser: -13.9±103.7, P=0.28	CMV: 8.5±11.6; nonuser: 2.5±10.8, P=0.08	CMV: -0.30±1.05 Nonuser: -0.07±0.99 *P=0.048	CMV: 4.2±7.3; nonuser: 5.5±7.8, P=0.33	Not mentioned
Smelt et al. [6]. After 48 months	CMV: 2.33±0.09; nonuser: 2.36±0.08, P=0.11	CMV: 96.4±117.0; nonuser: 97.0±96.1, P=0.97	CMV: 25.2±10.0; nonuser: 19.3±9.1, *P=0.041	CMV: 8.3±0.9 Nonuser: 8.5±0.8 P=0.09	CMV: 16.3±6.0; nonuser: 16.6±6.4, P=0.73	Not mentioned
Heusschen et al. [33]. Δ0-12 months	Optimum 1.0: +0.05±0.11 Optimum 2.0: +0.05±0.09 P>0.05	Optimum 1.0: +8.1±55.4; Optimum 2.0: +3.5±54.0, P>0.05	Optimum 1.0: +5.1±9.2; Optimum 2.0: +4.8±13.5, P>0.05	Optimum 1.0: -0.2±0.6 Optimum 2.0: -0.3±0.6 P>0.05	Not mentioned	Optimum 1.0: +0.03±0.07; Optimum 2.0: +0.01±0.06, P>0.05
Heusschen et al. [33]. After 12 months	Optimum 1.0: 2.40±0.08; Optimum 2.0: 2.39±0.09, P>0.05	Optimum 1.0: 139.4±104.7; Optimum 2.0: 124.1±101.8, P>0.05	Optimum 1.0: 21.8 ±10.0; Optimum 2.0: 19.7±13.4, P>0.05	Optimum 1.0: 8.4±0.7 Optimum 2.0: 8.4±0.8 P>0.05	Not mentioned	Optimum 1.0: 0.83±0.06; Optimum 2.0: 0.81±0.06, P>0.05

**Table 11:** Details of the selected studies. Data presented as mean±SD. *P≤0.05 or P<0.01 or P<0.001. CMV, customized multivitamin; PTH, parathyroid hormone; SMV, standard multivitamin; SD, standard deviation

Study	PTH (pmol/L)	Vitamin B1 (nmol/L)	Vitamin B6 (nmol/L)	Vitamin B_12_ (pmol/L)	Vitamin D (nmol/L)	Zinc (mcmol/L)	Others
Normal values	1.3-6.8	95-175	25-100	150-640	>50	9.2-18.4	
Dogan et al. [16]. Δ0 – 12 months	SMV: +1.26±2.39; CMV: +1.75±3.1, P=0.31	SMV: -7.05±30.1; CMV: -11.2±35.4, P=0.47	SMV: +28.0±36.8; CMV: +39.2±58.1, P=0.20	SMV: -38.9±141.3; CMV: +44.1±138.8, *P=0.002	SMV: +33.0±27.2; CMV: +25.0±27.0, P=0.09	SMV: +0.35±3.38; CMV: +1.36±2.44, P=0.06	
Dogan et al. [16]. At 12 months	SMV: 4.81±2.4; CMV: 5.80±3.4, *P= 0.052	SMV: 147.3±31.8; CMV: 151.2±31.3, P=0.49	SMV: 96.0±37.5; CMV: 111.5±57.8, P=0.07	SMV: 267.2±100.1; CMV: 349.8±122.1, *P<0.001	SMV: 76.7±24.6; CMV: 70.4±25.4, P=0.92	SMV: 12.31±3.12; CMV: 12.52±2.20, P=0.66	
Homan et al. [34]. After 3 years	SMV: 5.5±0.3; CMV: 5.0±0.3; none: 5.4±0.6, P>0.05	SMV: 150±3; CMV: 154±3; none: 128±6, P (CMV vs. SMV)>0.05, *P (CMV vs. nonuser)<0.001, *P (SMV vs. nonuser)=0.002	SMV:90±18; CMV:119±16; none:85±39, P>0.05	SMV: 264±12; CMV: 335±12; none: 290±26, *P (CMV vs. SMV)<0.001, P (CMV vs. nonuser)>0.05, P (SMV vs. nonuser)>0.05	SMV: 77±3; CMV: 81±3; none: 56±5, P (CMV vs. SMV)>0.05, *P (CMV vs. nonuser)<0.001, *P (SMV vs. nonuser)=0.002	SMV: 12.1±0.3; CMV: 12.8±0.3; none:10.8±0.5, P (CMV vs. SMV)=0.137, *P (CMV vs. nonuser)<0.003, P (SMV vs. nonuser)=0.098	
Perin et al. [32]. After 6 months	PTH (pg/mL), normal range: 10-65; SMV: 37.0±10.4; CMV: 27.0±14.4, P=0.547	Thiamine (nmol/L), normal range: 66.5-200 24; SMV: 111.4±27; CMV: 138.8±48.2, P=0.198	Not measured	Vitamin B12 (pg/mL), normal range: 200-900; SMV: 670.0±358.1; CMV: 904.5±341.2, P=0.244	Vitamin D (ng/mL), normal range: 32-100; SMV: 32.0±11.4; CMV: 40.3±18.9, *P=0.050	Not measured	Vitamin E-α (mg/L), normal range: 5.7-19.9; SMV: 7.5±2.6; CMV: 10.4±3.2, *P=0.050
Schijns et al. [5]. Δ0-3 years	CMV: -0.1±2.7; nonuser: +1.4±3.0, *P≤0.05	Not measured	Not measured	CMV: +59±191; nonuser: -2±146, *P≤0.05	CMV: +54±31; nonuser: +43±28, *P≤0.05	Not measured	
Schijns et al. [5]. After 3 years	CMV: 4.4±2.6; nonuser: 5.2±3.3, *P≤0.05	Not measured	Not measured	CMV: 372±175; nonuser: 296±138, *P≤0.05	CMV: 96±27; nonuser: 84±32, *P≤0.05	Not measured	Lisanne et al. [15]. After 3 years
Heusschen et al. [17]. Δ0-12 months	SMV: -0.10±2.66; CMV: -0.32±1.90, P>0.05	SMV: -14.02±43.87; CMV: -20.97±46.62, P>0.05	SMV: +5.85±30.77; CMV: +3.06±26.59, P>0.05	SMV: -29.30±83.69; CMV: -25.27±83.19, P>0.05	SMV: +53.77±25.53; CMV: +48.24±28.22, P>0.05	SMV: -0.81±2.15; CMV: -0.44±2.25, P>0.05	
Heusschen et al. [17]. After 12 months	SMV: 4.0±2.1; CMV: 3.2±1.7, *P≤0.05	SMV: 146.2±44.4; CMV: 146.9±33.2, P>0.05	SMV: 78.2±25.9; CMV: 82.9±27.3, P>0.05	SMV: 286.0±87.6; CMV: 277.5±77.8, P>0.05	SMV: 86.9±27.7; CMV: 88.0±28.4, P>0.05	SMV: 11.8±1.7; CMV: 11.7±1.9, P>0.05	
Smelt et al. [6]. Δ0-48 months	CMV: -0.8±4.1; nonuser: -0.4±3.5, P=0.58	CMV: 12.5±40.2; nonuser: 14.7±37.6, P=0.75	CMV: 8.0±126.9; nonuser: 23.0±52.9, P=0.42	CMV: 60.2±176.7; nonuser: 78.7±216.3, P=0.69	CMV: 23.5±30.4; nonuser: 24.7±27.9, P=0.82	Not mentioned	
Smelt et al. [6]. After 48 months	CMV: 5.9±2.2; nonuser: 6.5±2.6, P=0.21	CMV: 159.0±100.8; nonuser: 150.2±33.3, P=0.54	CMV: 98.0±48.8; nonuser: 101.1±39.9, P=0.70	CMV: 381.1±227.7; nonuser: 363.0±174.9, P=0.62	CMV: 65.7±21.1; nonuser: 67.3±21.8, P=0.69	Not mentioned	
Heusschen et al. [33]. Δ0-12 months	Optimum 1.0: -0.3±2.1; Optimum 2.0: +0.4±1.6, P>0.05	Optimum 1.0: -19.3±40.6; Optimum 2.0: -14.6±37.5, P>0.05	Optimum 1.0: +3.1±26.6; Optimum 2.0: +25.7±29.7 *P≤0.05	Optimum 1.0: -32.9±76.2; Optimum 2.0: +5.5±103.7, *P≤0.05	Optimum 1.0: +48.8±29.0; Optimum 2.0: +28.6±23.4, *P<0.001	Optimum 1.0: -0.4±2.2; Optimum 2.0: +1.3±3.9, *P≤0.05	
Heusschen et al. [33]. After 12 months	Optimum 1.0: 3.5±1.9; Optimum 2.0: 3.5±2.0, P>0.05	Optimum 1.0: 145.4±29.8; Optimum 2.0: 153.9±36.5, P>0.05	Optimum 1.0: 82.9±27.3; Optimum 2.0: 99.8±31.7, *P≤0.05	Optimum 1.0: 267.3±80.0; Optimum 2.0: 302.4±93.2, *P≤0.05	Optimum 1.0: 84.5±32.3; Optimum 2.0: 86.2±22.5, P>0.05	Optimum 1.0: 11.7±1.9; Optimum 2.0: 12.6±2.1, *P≤0.05	

Vitamin B_12_

Dogan et al. found that after 12 months, the serum levels of vitamin B_12_ in the SMV group decreased, but in the WLS Forte group, the levels increased (P<0.001) [16]. At three years follow-up of the same patients’ groups, vitamin B_12_ deficiency was lower in the WLS Forte group compared to the SMV group (P<0.001) [34]. Another cohort study has shown similar results [5]. A year post-RYGB surgery, the mean serum concentration of vitamin B_12_ was noted to be 347.3±145.1 pmol/L in the WLS Forte group versus 276.8±131.4 pmol/L in the SMV group (P<0.001) [5].

The significant difference between both groups remained unchanged after three years of follow-up (P=0.028) [5]. The chewable CMV resulted in no significant difference (P=0.271) between the investigational group and the standard group in vitamin B_12_ deficiency after three and six months, both groups were within the normal range [32]. 

In the initial trial by Heusschen et al. on Opt. 1.0 in SG patients, it was found that there was no significant difference in the deficiencies between both groups [17]. Vitamin B_12_ in the WLS Optimum group decreased by an average of 32.93±76.25 pmol/L, while in the SMV it decreased by 34.17±91.11 pmol/L [17]. When Opt.1.0 was refined to be Opt. 2.0 and both versions were compared, vitamin B_12_ serum levels increased in the latter version and decreased in opt.1.0 (P=0.18) [33]. The deficiency in opt. 2.0 was lower than 1.0 (P=0.031) [33]. 

In a five years cohort study, WLS Opt. 1.0 prevented vitamin B_12_ deficiencies during the first year (P=0.045) [6]. Gradually starting month 24, both groups SMV and WLS showed a similar percentage of newly discovered deficiencies reaching 9.5% WLS Opt. 1.0 vs. 5.6% SMV (P=1.00) by the 48th month [6]. 

Vitamin D, parathyroid hormone, and calcium

WLS Forte Opt. 1.0 and 2.0 do not contain calcium; hence, external supplementation of calcium was given [5,6,16,17,33,34]. Extra 1200 IU of vitamin D and 1500 mg of calcium were supplemented in Dogan et al. study with SMV and WLS Forte [16]. This led to insignificant differences in both groups in the serum concentration of vitamin D as well as in the deficiency (P=0.87, P=0.168) [16]. The same was noticed for PTH and calcium levels (P>0.05) [16]. On a longer term of three years, the results remained consistent for all three elements: vitamin D, PTH, and calcium, when WLS Forte was compared to SMV (P>0.05) [34]. However, when vitamin D deficiencies were compared in the WLS Forte group to nonusers (P<0.001) and SMV group to nonusers (P<0.002), the difference was significant in favor of WLS Forte and SMV [34]. 

After three years in the larger-scale cohort study, where the intake of calcium supplementation of 500 mg and vitamin D of 880 IU was advised, the serum levels of vitamin D were significantly higher in the WLS Forte group compared to the nonuser group (P=0.029) [5]. Higher PTH serum levels were noticed in the nonuser group compared to the WLS Forte group (P=0.004) [5].

In the trial studying the chewable CMV, an intake of 600 IU of vitamin D and 1800 mg of calcium was advised [32]. The vitamin D serum levels were found to be significantly higher in the investigational group (P=0.033) and PTH was significantly lower (P=0.042) [32]. 

In Opt. 1.0, the deficiencies and serum levels of vitamin D and PTH were similar in the test group and control group (P>0.05) [6,17]. The increase in vitamin D and the decrease in PTH levels were better with Opt. 1.0 when compared to Opt. 2.0, however, still not significant [33]. Calcium deficiency was rarely found as a result of external supplementation of 1500 mg calcium a day [6,17,33].

Iron, hemoglobin, ferritin, and folic acid

Dogan et al. found that at baseline, the mean hemoglobin serum levels were 8.5±0.7 mmol/L and 8.6±0.7 mmol/L (P=0.24) for SMV and WLS Forte, respectively [16]. These values remained the same for the trial period of 12 months [16]. In the SMV group, the mean serum ferritin was reduced by 18.4±61.8 mg/L, but in the WLS Forte group, it remained consistent (P=0.08) [16]. The folic acid serum levels were increased significantly in the WLS Forte participants at six and 12 months when compared to SMV participants (P≤0.05) [16]. Homan et al. study that lasted for three years found that the estimated mean serum concentration for hemoglobin was considerably greater in the WLS Forte group in comparison to SMV users (P=0.05) and nonusers (P=0.003) [34]. When comparing the WLS Forte group to the SMV group and the nonuser group, the mean serum ferritin concentration was greatly higher in the WLS Forte group (P=0.001, P=0.047) [34]. The same was noticed in folic acid serum levels (P<0.001 for both) [34]. Another cohort study showed similar results, where serum ferritin levels and folic acid levels were higher in the WLS Forte group compared to nonusers (P=0.016 and P<0.001) [5]. All three elements, iron, iron-binding capacity, and iron saturation, did not show significant differences in the serum levels or deficiencies when the investigational multivitamin in Perin et al. study was compared to SMV (P=0.638, 0.107, and 0.065, respectively) [32]. Generally, iron and iron saturation were deficient in both participants but not iron-binding capacity [32].

In contrast to SMV, the Smelt et al. study showed that Opt. 1.0 resulted in elevated levels of iron deficiency, but when both groups were compared it was an insignificant difference (P>0.05) [6]. Similarly, in relation to ferritin levels, the difference between both groups was not significant (P>0.05); still, the overall decrease was greater in the SMV group [6]. Folic acid serum levels were higher in Opt. 1.0 (P≤0.05) [6].

Hemoglobin levels in Opt. 2.0 were consistent with Opt. 1.0, and both groups showed lower levels of hemoglobin [17,33]. The increase in the levels of ferritin and folic acid in the Opt. 1.0 group was higher than Opt. 2.0 [17,33].

Dogan et al. found that at baseline, the mean hemoglobin serum levels were 8.5±0.7 mmol/L and 8.6±0.7 mmol/L (P=0.24) for SMV and WLS Forte, respectively [16]. These values remained the same for the trial period of 12 months [16]. In the SMV group, the mean serum ferritin was reduced by 18.4±61.8 mg/L, but in the WLS Forte group, it remained consistent (P=0.08) [16]. The folic acid serum levels were increased significantly in the WLS Forte participants at six and 12 months when compared to SMV participants (P≤0.05) [16]. Homan et al. study that lasted for three years found that the estimated mean serum concentration for hemoglobin was considerably greater in the WLS Forte group in comparison to SMV users (P=0.05) and nonusers (P=0.003) [34]. When comparing the WLS Forte group to the SMV group and the nonuser group, the mean serum ferritin concentration was greatly higher in the WLS Forte group (P=0.001, P=0.047) [34]. The same was noticed in folic acid serum levels (P<0.001 for both) [34]. Another cohort study showed similar results, where serum ferritin levels and folic acid levels were higher in the WLS Forte group compared to nonusers (P=0.016 and P<0.001) [5]. All three elements, iron, iron-binding capacity, and iron saturation, did not show significant differences in the serum levels or deficiencies when the investigational multivitamin in the Perin et al. study was compared to SMV (P=0.638, 0.107, and 0.065, respectively) [32]. Generally, iron and iron saturation were deficient in both participants but not iron-binding capacity [32].

In contrast to SMV, the Smelt et al. study showed that Opt. 1.0 resulted in elevated levels of iron deficiency, but when both groups were compared it was an insignificant difference (P>0.05) [6]. Similarly, in relation to ferritin levels. the difference between both groups was not significant (P>0.05); still the overall decrease was more in the SMV group [6]. Folic acid serum levels were higher in Opt. 1.0 (P≤0.05) [6].

Hemoglobin levels in Opt. 2.0 were consistent with Opt. 1.0, and both groups showed lower levels of hemoglobin [17,33]. The increase in the levels of ferritin and folic acid in the Opt. 1.0 group was higher than Opt. 2.0 [17,33].

Vitamins B1 and B6

In RYGB patients, vitamin B1 serum levels showed no significant difference between the WLS Forte group and SMV group (P>0.05) in two studies [16,34]. Meanwhile, the values were within normal ranges [16,34]. The difference was significant in vitamin B1 serum levels when the WLS Forte group was compared to nonusers (P<0.001) and the SMV group to nonusers (P<0.002) [34]. Vitamin B6 has a normal serum level that ranges between 25 and 100 nmol/L. Vitamin B6 serum levels were more elevated in the participants in the WLS Forte group (111.5±57.8 nmol/L) than the ones in the SMV group (96.0±37.5 nmol/L) (P=0.036) [16]. After three years the same was observed, WLS Forte group's (119±16 nmol/L) vitamin B6 levels were higher than SMV group (90±18 nmol/L) (P>0.05) [34]. In the chewable CMV group, the vitamin B1 levels were significantly better than those in the SMV group (P<0.009) [32].

In SG patients, Opt. 1.0 showed substantially greater vitamin B1 serum levels compared to SMV in two studies: 148.0±27.6 nmol/L vs. 134.8±24.8 nmol/L (p=0.011) [6,17]. In the Smelt et al. study, after five years, the CMV group showed elevated vitamin B6 that was close to the upper limit (98.0±48.8 nmol/L) and the nonuser group went beyond the upper limit (101.1±39.9 nmol/L) [6].

A study comparing Opt. 1.0 and 2.0 discovered that the serum level of vitamin B6 was higher in 2.0 (99.8±31.7 nmol/L) than in 1.0 (82.9±27.3 nmol/L) (P=0.014); in vitamin B1 the difference was minimal (P>0.05) [33].

Zinc and magnesium

There was no significant difference noticed in zinc and magnesium serum levels among groups in the two studies (P>0.05), and normal levels were maintained [16,33]. A cohort study indicated that serum levels of zinc were significantly higher in the WLS Forte group when compared to the nonuser group (P<0.003), but not higher when SMV compared to the nonuser (P=0.098) [5].

Zinc levels in the Opt. 2.0 group increased after 12 months, while it declined in the Opt. 1.0 group (+1.33.9 mol/L vs. -0.42.2 mol/L, P=0.001) [16,17,33]. There was no difference in the prevalence of zinc deficiency after 12 months between Opt. 1.0, 2.0, and SMV (P>0.05 for both) [6,7,33]. Magnesium serum levels were better with Opt. 1.0 when compared to 2.0; however, the difference was not significant, and both retained normal levels (P>0.05) [33].

Weight loss

The relationship between weight loss and vitamin and mineral deficiency was an important consideration. Two studies correlated the weight loss achieved by RYGB patients following the ingestion of WLS Forte and SMV [16,34]. The trial indicated that after 12 months, the weight loss in both groups was almost similar (P=0.24) [16]. Although, over a longer period of three years, the weight loss in the WLS Forte group was higher than the SMV and the nonuser groups (P≤0.05, P=0.040) [34].

In SG patients, the duration of the trials that studied weight loss was one year and both showed similar weight loss in all groups: Opt. 1.0, 2.0, and SMV [17,33].

Adherence

The nonusers (stopped ordering) of WLS Forte were 20% [34]. In another two studies, one-third of the patients were non-compliant to Opt. 1.0 and 55% were non-compliant to Opt. 1.0 and 2.0 mainly due to reported nausea [30,34].

Discussion

The aim of the review was to establish if CMV would result in fewer deficiencies and higher serum levels in adults’ post-bariatric surgery when compared with SMV treatment. The results of this systemic review supported that CMV and SMV were almost similar in maintaining the serum levels of vitamins and minerals.

Six of the presented studies advocated the intake of CMV over SMV for different reasons, but this was not justified by the results [5,6,16,32-34]. The serum levels of all vitamins and minerals in the Perin et al. study were not promising in relation to SMV except for vitamin D and B1 [32]. Also, the chewable CMV caused a deficiency in the iron and iron saturation serum levels still the researchers supported the CMV intake. The justification was that the chewable CMV provided better palatability, increased bioavailability, and reduced the number of pills taken by patients: vitamin B_12_ and vitamin D in this case [32]. Enhancement of the palatability of the CMV by providing a chewable alternative would help with adherence, as one of the concerns of the patients was that the tablet is hard to swallow [22]. The content of vitamin B_12_ in the chewable CMV (500 mcg) was lower than the SMV (512 mcg); however, the serum level in the investigational group was much higher due to the increased bioavailability (904.5±341.2 pg/mL vs. 670.0±358.1 pg/mL) [32]. Chewable tablets would be a suitable substitute for vitamin B_12_ injections recommended by the BOMSS [14].

Heusschen et al. thought that Opt. 1.0 was unable to provide a clear benefit to patients over SMV [17]. Opt. 1.0 showed significant improvement in folic acid and PTH levels only, while all other elements were of comparable levels with SMV [17].

Regardless of the results that were coherent with Heusschen et al., Smelt et al., who studied Opt. 1.0 for five years, disputed that the CMV would be a better option when compared to SMV [6,17]. The cohort study did not support the dispute as only folic acid serum levels were significantly improved [6]. It presented that vitamin B6 levels in both groups were closer to or beyond the upper limit of the normal serum level range, which indicates susceptibility to hypervitaminosis. Hypervitaminosis due to vitamin B6 that manifests in neurological symptoms was reported as a common occurrence that could reach 50% post-bariatric surgeries due to multivitamins intake [35].

The same dose of 2 mg of vitamin B6 was incorporated in Opt. 2.0, however, switched pyridoxine that was in Opt. 1.0 with pyridoxal-phosphate in Opt. 2.0 as it is known to cause less toxicity [33,36]. When Opt. 2.0 was compared to 1.0, Heusschen et al. revealed high levels of vitamin B6 (99.8±31.7 nmol/L) for Opt. 2.0 within shorter time of one year [33].

In Opt. 2.0, Heusschen et al. applied the modification in vitamin B6 and increased the doses of iron, folic acid, vitamins B_12_, B1, and zinc [17,33].

The iron deficiency rate has decreased, and folic acid serum levels were higher with Opt. 1.0 when compared to SMV and Opt. 2.0 [6,17,33]. This was inconsistent with the concentration of iron and folic acid found in Opt. 1.0 and 2.0 [33]. When a per-protocol analysis was done, it was found that 3% of the Opt. 2.0 group was deficient vs. 3% of group 1.0 indicating that the reason for the discrepancy in the results was non-compliance [33]. This suggests that the dose of iron in Opt. 2.0 of 28 mg would be sufficient to lower deficiencies in SG patients and lower the risk of GIT side effects [6,17,33]. In Opt. 2.0, the dose of 100 mcg was sufficient to prevent vitamin B_12_ deficiencies in SG patients [13,33]. Contrary to the ASMBS advice of 45-60 mg of iron and 300 to 500 mcg of vitamin B_12_ [11,33]. The fact that some women at menstruating age would need more iron supplementation would require attention in future studies. Vitamin B1-increased dose from 2 mg to 2.75 mg led to an increase in the cases of hypervitaminosis by 35%, but the incidence of intoxication is highly unlikely [19,33,37].

In RYGB, WLS Forte improved vitamin B_12_, ferritin, and folic acid serum levels compared to SMV. Iron, vitamin B1, zinc, and magnesium serum levels were unchanged and did not present in deficiencies [5,16,34]. This indicates that the concentrations of these vitamins and minerals in the WLS Forte were adequate to avoid deficiency in this group of patients; refer to Table 9 for the concentration of each vitamin.

Vitamin D, hemoglobin, and PTH results were conflicting; one study mentioned that there was a significant difference between WLS Forte and SMV, while the other mentioned that there was an insignificant difference [5,16,34]. However, the studies that reported significant differences were the longer ones, which might imply the reason for the change [5,34].

The concentration of vitamin B6 in the WLS Forte and SMV were 2.80 and 1.40 mg, respectively [16,34]. Vitamin B6 hypervitaminosis was reported in the group consuming WLS Forte and was borderline in the group consuming SMV [16,34]. Dogan et al. advised that the vitamin B6 dose in WLS Forte has been reduced to 0.98 mg [16].

Although three researchers supported WLS Forte and have proved the enhanced ferritin, folic acid, and vitamin B12 serum levels with the CMV, WLS Forte did not contain calcium, and an external supplementation of calcium/vitamin D was given [5,16,34]. WLS Forte was reported to be more expensive than SMV by 1.6 times [5]. If the cost of WLS Forte was higher and external supplementation was required in both WLS Forte and SMV, patients would be tempted to purchase SMV [22]. Patients would then ask their doctors to advise on the external supplementation required to replenish any other prevalent deficiency.

Recommended modifications in CMV

Heusschen et al. recommended improvement in Opt. 2.0 formula to contain 1.5 mg of pyridoxal-phosphate instead of 2 mg to avoid vitamin B6 hypervitaminosis [33]. Since WLS Forte, chewable CMV, Opt. 1.0 and 2.0, had no calcium; another suggestion would be to integrate a dose of 1500 mg of calcium and a total dose of 3000 IU of vitamin D [11,13]. Boyce et al. confirmed that the separation of calcium and iron was not mandatory [20]. Thus, the addition of calcium would help in simplifying the protocol followed by the patients and increase adherence. The iron content in the chewable CMV for the RYGB would need to be increased from 36 mg to at least 45 mg as advised by the ASMBS [11,13]. Changing the formula to an easier-to-swallow or chewable tablet, also, helps with adherence [22].

The use of the CMV would not be recommended in clinical practice until these modifications are reviewed. It would be advised to continue to take SMV along with external supplementation in case any would be required.

Strengths and limitations

A major strength would be that three trials were double-blinded and randomized and the included cohort studies had longer follow-up duration [5,6,16,17,32,34].

However, the four included trials had short follow-up periods [16,17,32,33]. Thus, the impact of longer-term deficiency and post-surgery complications was not reflected. The dropout rate was so high that it reached 45% by the sixth month in the Perin et al. study and was high in the Smelt et al. study too [6,32]. Meanwhile, the lost population was similar across both arms [6,32]. This dropout rate could have impacted the results as much as the limited number of participants could have, as they might have resulted in statistical significance in the outcomes. Most of the studies were focused on the Netherlands and only one in the United States [5,6,16,17,32,33,34].

Preoperative vitamin and mineral deficiencies were not treated in most of the studies, except for vitamins B_12_ and D in four studies; but patients who used additional supplementation were excluded from serum level data to avoid biased estimates and reflect accurate results [5,6,7,16,17,23,24,32-34]. In all these studies, there were no data on dietary consumption, which could have affected the vitamin and mineral serum levels.

Compliance and measurement of multivitamin consumption was a key consideration. All of the studies contained a self-reported parameter that depended on the accurate reporting and honesty of the subjects [5,6,16,17,32-34]. In some studies, individuals were blinded to the nature of the supplement; hence it is possible that non-compliant patients were evenly distributed between the two groups [16,34]. In the Schijns et al. study, the range of different supplements utilized by the 258 nonusers had wide vitamin concentrations that were not consistent among the group and could have impacted the results [5].

## Conclusions

WLS Forte results were better than SMV and placebo. WLS Forte lowered deficiencies in RYGB patients in vitamins B_12_, vitamin D, hemoglobin, folic acid, and ferritin. In the chewable CMV, the serum levels of many essential vitamins were similar to those of SMV. Only vitamin D and B1 levels were better in the CMV than in the SMV, but iron levels were deficient. For SG patients, Opt. 1.0 improved folic acid initially and PTH serum levels when compared to SMV. Opt. 2.0 results were relatively better than Opt. 1.0. The optimized version Opt. 2.0 presented superior serum levels of vitamins B_12_ and zinc but comparable folic acid levels. Further modifications in the present CMV formulas would be required. These improvements would include the incorporation of calcium, reduction in vitamin B6, and modifying the formula to be easier to swallow. The modifications should simplify the protocol to be one multivitamin tablet or a maximum of two a day. In conclusion, the use of the CMV would not be recommended in clinical practice until they are modified and tested further. For now, it would be recommended to maintain the consumption of SMV along with external supplementation in case any would be required. Regular monitoring by a doctor is recommended to check the vitamin and mineral serum levels to avoid deficiency or intoxication.

Future research recommendations

Future studies should treat preoperative deficiencies of vitamins and minerals and analyze the impact of the dietary consumption of the participants. A precise measure of compliance should be validated and used. Multi-center trials that monitor the effect of the modified CMV on the serum levels of vitamins and minerals for the longer term in different wider populations are needed. This would establish if the results reported in the current study are applicable. Studying toxicity that would occur in the longer term, especially with vitamin B6 and any other issues such as financial or side effects would be of benefit. The studies included in this review did not investigate if the rate of weight loss was impacted by vitamin supplementation. Weight loss and its relation to vitamin supplementation would be important to measure in future randomized controlled trials.
